# Altered System-Level Integration Between Working Memory and Gaze Dynamics in Children Treated for Posterior Fossa Tumors

**DOI:** 10.11621/pir.2026.0105

**Published:** 2026-03-25

**Authors:** Sofia A. Mironets, Victoria I. Ismatullina, Timofey V. Adamovich, Sergey B. Malykh

**Affiliations:** a *Dmitry Rogachev National Medical Research Center of Pediatric Hematology, Oncology, and Immunology, Moscow, Russia*; b Federal Scientific Center for Psychological and Interdisciplinary Research, Moscow, Russia

**Keywords:** cerebellum, posterior fossa tumor, movements, working memory, visual-spatial ability, reading

## Abstract

**Background:**

In recent years, experimental studies concerning the role of the cerebellum in cognitive functions and a variety of language deficits have been conducted.

**Objective:**

To evaluate the impact of posterior fossa tumors (PFT) on spatial working memory and eye-movement behavior during reading in children and to compare the system-level organization of working memory and gaze-related parameters between patients and typically developing peers.

**Design:**

This retrospective cohort study applied advanced analytical methods, including Drift Diffusion Modeling (DDM) and Recurrence Quantification Analysis (RQA), to capture the temporal dynamics of cognitive processing and oculomotor control. The study included 119 children aged 8 to 17 years: 59 survivors of posterior fossa tumors and 60 healthy controls. Working memory was assessed using the Spatial Span task from the CANTAB battery, modeled using DDM parameters, while eye movements during reading were recorded with eye tracking and analyzed using RQA. Network analysis was conducted to characterize cross-domain integration between working memory dynamics and gaze behavior.

**Results:**

Our findings indicate that cerebellar disruption due to tumor and treatment affects not only isolated aspects of reading or eye-movement behavior, but also the functional coordination between working memory and oculomotor control. Stronger working memory capacity was associated with more organized and predictable gaze dynamics in controls, whereas patients showed reduced flexibility and altered connectivity patterns. RQA parameters captured a unified dynamical system, linking cognitive control and motor implementation during reading.

**Conclusion:**

These findings highlight the importance of considering subtle, system-level disruptions in posterior fossa tumor patients and underscore the relevance of using multimodal, temporally sensitive approaches when designing rehabilitation protocols.

## Introduction

Cognitive functioning is a complex system of mental processes that enable the perception, storage, processing, and utilization of environmental information. Two critical components of this system are visual perception and working memory, whose interaction is essential for goal-directed behaviors, including reading (Kofler et al., 2019). Visual perception initiates sensory selection and preliminary analysis of visual stimuli, while working memory maintains and manipulates this information to guide ongoing tasks and behavioral responses (Baddeley, 2007). Such interaction between visual perception and working memory is fundamental for reading, an intricate cognitive activity that demands synchronized functioning of visual, mnemonic, and executive systems (Peng et al., 2018; Sarver et al., 2012). The cerebellum, traditionally recognized for its role in motor coordination, has increasingly been shown to contribute significantly to cognitive functions such as attention, working memory, decision-making, and oculomotor control (Bellebaum & Daum, 2007; Koziol et al., 2012). Robust evidence points to specific cerebello-cortical connections activated during cognitive tasks, emphasizing the cerebellum’s integral role in higher-order processing beyond purely motoric functions (Pelzer et al., 2017). Cerebellar lesions, common among children surviving posterior fossa tumors (PFT), often lead to persistent cognitive and motor deficits, despite high survival rates (Cámara et al., 2020; Zilli et al., 2021). These deficits result primarily from surgical intervention, radiotherapy, and chemotherapy, which disrupt cortico-cerebellar pathways (Tanedo et al., 2022). Although cognitive and motor impairments in pediatric patients have been extensively studied, disturbances in reading—an essential skill crucial for academic success—have received comparatively little attention.

Reading difficulties following cerebellar lesions likely arise due to compromised control of eye movements, with specific cerebellar regions playing critical roles in oculomotor functioning (Beh et al., 2017). Key reading-related oculomotor characteristics, such as fixation duration, the number of fixations, and frequency of regressive saccades, may be disrupted in individuals with cerebellar pathology (Litovchenko, 2012; Rimrodt et al., 2009; Tomaiuolo et al., 2021). Additionally, the interplay between visual-spatial working memory and oculomotor control during reading underscores the integration of motor and cognitive functions. Hence, cerebellar lesions may induce impairments not merely at isolated perceptual or motor levels, but also at integrative, system-level interactions between cognition and eye movement control, potentially affecting broader cognitive outcomes, including academic achievement.

Given that reading and working memory depend on coordinated interactions across multiple cognitive and oculomotor subsystems, alterations following cerebellar injury may be better understood by analyzing system-level organization rather than isolated behavioral outcomes.

Standard neuropsychological assessments often overlook subtle yet impactful network-level disruptions in cognitive architecture. Therefore, understanding how cognitive processes interact—not just their isolated performance—is essential. To address this gap, the current study employs a system-level approach, integrating advanced analytical methods such as Drift Diffusion Modeling (DDM), Recurrence Quantification Analysis (RQA) of eye movements, and network modeling (Anderson et al., 2013; LaCombe & Barenholtz, 2015; Tschense & Wallot, 2020). Drift Diffusion Modeling is a computational framework used to decompose decision-making processes into underlying cognitive components such as evidence accumulation rate, response thresholds, and non-decision time, thereby providing insight into subtle cognitive changes not evident from behavioral data alone (Ratcliff & McKoon, 2008; Turner et al., 2016; Velichkovsky et al., 2020; Voronin et al., 2020; Zakharov & Ismatullina, 2021; Zhuikov, 2021). The model provides a rigorous mathematical framework that decomposes observed decision-making behavior into underlying cognitive processes. Initially developed to model simple two-choice decisions, DDM has since become a cornerstone methodology in cognitive psychology. It offers researchers insight into the latent mechanisms operating between stimulus presentation and response execution. In recognition memory paradigms, the model differentiates memory strength, represented by the drift rate, from response bias, captured by the starting point parameter. This clear dissociation has significantly enhanced our understanding of how factors such as aging, brain injury, and individual variability influence memory-based decision-making (Myers et al., 2022). In clinical practice, the DDM is used to study changes in cognitive processes associated with various disorders. For example, research has shown alterations in DDM parameters in patients with Parkinson’s disease, attention deficit hyperactivity disorder (ADHD), autism spectrum disorders, obsessive-compulsive disorder (OCD), and schizophrenia. The model helps to understand whether these patients’ behavioral difficulties are related to slowed information processing (drift rate), changes in decision strategy (“caution,” threshold width), or problems with motor output (non-decision time) (Gupta et al., 2022).

Recurrence Quantification Analysis (RQA) is a nonlinear analytical technique employed to quantify temporal patterns and complexity within gaze trajectories, capturing the dynamics and predictability of eye movements during reading (Anderson et al., 2013; Emel’ianova & Runnova, 2021; Kirichenko et al., 2016; Kiselev, 2006; Zhuravlev et al., 2023; Tschense & Wallot, 2020; Zhuykov, 2021).

Network modeling is widely used in medical psychology for analyzing complex interactions between symptoms, cognitive functions, and neurophysiological indicators (Andronova & Kapuza, 2025; Artemenkov, 2021; Shmukler et al., 2020; National Research University Higher School of Economics, n.d.). Network modeling complements these approaches by characterizing how cognitive and oculomotor variables are organized into interconnected systems, rather than isolated components. It enables the identification of integration strength, modular structure, and central nodes within the cognitive–motor network, providing a structural perspective on how cerebellar pathology may alter coordination across domains (Borsboom et al., 2021; Epskamp et al., 2018).

Employing these methods enables a fine-grained analysis of cognitive–motor integration and provides critical insights into the subtle disruptions associated with cerebellar pathology.

The primary goal of the present research is thus to elucidate how posterior fossa tumors and their treatment affect the dynamic interplay between working memory and eye-movement control during reading among pediatric patients. Specifically, we aim to:

1. Evaluate differences in visual-spatial working memory performance and decision-making parameters (DDM) between posterior fossa tumors (PFT) patients and healthy controls;
2. Compare eye-movement behaviors during reading tasks, particularly focusing on recurrence-based metrics of gaze stability and complexity (RQA), between the two groups;
3. Perform an exploratory network analysis to model the structure of cognitive–oculomotor relationships and reveal reorganizations associated with cerebellar injury.

We hypothesize that cerebellar disruption due to posterior fossa tumors results in measurable alterations of cognitive architecture, characterized by reduced integration between memory and motor systems, and changes in the dynamical complexity of eye-movement patterns. By modeling these interdependencies directly, the present study seeks to uncover sensitive markers of systemic cognitive disruption, informing both clinical assessment and targeted rehabilitation strategies.

## Methods

### Participants

The study included a total of 119 children aged between 8 and 17 years. Of this group, 59 children (*M* = 12.49 (2.5) y.o.; 44 % male) were treated for a posterior fossa tumor according to the HIT MED 2017 protocol, and went into remission successfully (patient group, PG). The other 60 children (*M* = 13.03 (2.67) y.o.; 47 % male) formed the control group (CG). The participants were native Russian speakers and had attended school for at least two years.

The PG included participants with tumors located in the brain and/or cerebellar hemisphere and fourth ventricle. All children had completed treatment, including surgery, chemotherapy, and were in remission. The duration of remission ranged from a minimum of 3 months to a maximum of 158 months (*M =* 47.37, *SD =* 37.22). The age at disease onset ranged from 3 months to 16 years (*M =* 6.98, *SD =* 3.4 years).

### Procedure

The assessment was conducted as a single-session procedure by licensed neuropsychologists. Patient evaluations were carried out by a clinical psychologist at the Russkoye Pole Clinical Rehabilitation Research Center of the Dmitry Rogachev National Medical Research Center of Pediatric Hematology, Oncology, and Immunology. All participants older than 15 years and the legal representatives of younger children gave written informed consent before the initial tests. All testing for the control group took place in a quiet room in the children’s school during school hours by trained research assistants. The CANTAB and reading tasks were administered in one session lasting approximately 30 minutes, with a 5-minute break between tasks to reduce fatigue.

#### Spatial Span (SSP)

We selected the Spatial Span (SSP) test to evaluate Spatial Working Memory. A computerized version of the Corsi blocks from Cambridge Neuropsychological Test Automated Battery (CANTAB) was used. In this task, the participant needs to memorize and reproduce the sequence of elements, the number of which increases from 2 to 9. The average testing time was about 5 minutes. Answers were recorded using a touch screen. Descriptions of the test parameters (Cambridge Cognition, n.d.; Downes et al., 2006) are presented in *Table 1*.

**Table 1:** Assessment Score from SSP Test and DDM Parameters

**Parameter**	**Meaning**
Span length	This is the longest sequence successfully recalled by the subject. The subject has three attempts at each level.
Total errors	The number of times the subject selected an incorrect box.
Number of attempts (span length n)	The number of attempts that the subject made for the span length, which can be set to a number from 2 to 9.
Mean time to last response (span length n)	The mean time the subject took to complete problems of the span length n (i.e., make n responses), which can be set for a number from 2 to 9. The time is measured from the end of the presentation phase (the moment the final box closes) to the time of the subject’s final response on a given attempt. Attempts undertaken on spans that the subject did not pass are included in this calculation.
Drift rate (v)	The speed and efficiency of evidence accumulation.
Decision boundary (a)	The threshold of accumulated evidence needed to make a response.
Non-decision time (t_0_)	The capturing processes unrelated to the decision itself, such as stimulus encoding and motor execution.

To characterize the temporal dynamics of behavior in the SSP task, we employed a drift diffusion model (DDM), a class of sequential sampling models traditionally used to describe decision-making in two-alternative forced-choice paradigms (Bogacz et al., 2006). In its classical formulation, the DDM models the accumulation of noisy evidence over time until a decision boundary is reached (Myers et al., 2022; Voronin et al., 2020; Voss et al., 2015).

In the present study, however, the SSP task does not consist of isolated binary decisions, but rather of extended action sequences that depend on an internal representation maintained in working memory. Accordingly, we adopted an extended, episode-level interpretation of the DDM, in which each behavioral sequence was treated as a single decision episode. Under this formulation, the model captures the integrated dynamics of behavior across the entire sequence, rather than modeling individual step-wise choices.

Within this framework, the DDM parameters were interpreted as follows. The drift rate reflects the efficiency of information accumulation over the course of the sequence, incorporating all sources of information available to the participant, including those related to the internal representation of the sequence. The decision boundary represents the response criterion or degree of caution, corresponding to the level of accumulated information required for committing to or completing the behavioral episode. The non-decision time accounts for processes not directly related to evidence accumulation, such as perceptual encoding and motor execution.

Importantly, this application should be understood as a phenomenological parameterization of behavior at the level of the full sequence, rather than a mechanistic model of individual step-level decisions. We do not assume that the DDM captures the internal microstructure of sequential choices within the SSP task.

This approach is consistent with broader perspectives on sequential sampling models as general frameworks for evidence accumulation (Forstmann et al., 2016; Gold & Shadlen, 2007), with interpretations of decision boundaries as reflecting optimal stopping or control criteria (Drugowitsch et al., 2012), and with prior work demonstrating that evidence accumulation can extend across temporally structured sequences rather than being confined to isolated trials (Nguyen et al., 2019).

DDM parameters were estimated for each participant using the PyDDM Python package (Shinn & Lam, 2018), based on individual reaction time data and binary response outcomes (*Table 1*).

#### Reading Task

We used seven grammatically simple texts (36–65 words) taken from a Russian-language textbook recommended by the Ministry of Education of the Russian Federation for elementary schools. Children read the excerpts presented on the screen, and two questions were asked after each text to ensure they understood the meaning. Participants read at their own pace to avoid mistakes.

Oculomotor function was recorded every 1/60 s monocularly by an Arrington eye-tracking system (Arrington Research Inc., Scottsdale, Arizona, USA) with chin support. The stimulus was displayed on the monitor (Samsung, 23", resolution 1920 × 1080 pixels) with a distance of 60 cm. The center of vision is determined by locating the center of the pupil. Calibration was conducted using the standard nine-point algorithm.

Eye-tracking data were preprocessed using a custom Python pipeline. Raw gaze recordings were segmented into text-related intervals and smoothed with a Savitzky–Golay filter. Eye-tracking data were analyzed using Recurrence Quantification Analysis (RQA), a nonlinear dynamical approach providing detailed characterization of gaze trajectory complexity. RQA metrics were computed using the pyunicorn library (Donges et al., 2016). Specific RQA parameters included recurrence rate (RR, proportion of gaze positions revisited), laminarity (LAM, gaze stability in specific screen regions), and entropy of diagonal lines (ENT, complexity of gaze pattern sequences). RQA parameters were calculated separately for each reading segment (each text) and then aggregated as mean and standard deviation values across segments to capture both general gaze behavior and variability across reading tasks.

### Statistical Analysis

To examine associations among eye-movement metrics, recurrence quantification analysis (RQA) measures, spatial span performance, and diffusion decision model (DDM) parameters, we conducted Spearman rank-order correlation analyses, suitable for non-parametric data and potential outliers. P-values were adjusted for multiple comparisons using the Holm correction. To assess differences between pediatric patients and healthy controls, we used analysis of covariance (ANCOVA), controlling for age and gender. Effect sizes for group comparisons were estimated using eta-squared (η^2^) to quantify the proportion of variance explained by each factor.

We used Gaussian Graphical Models (GGM) with graphical lasso regularization (Friedman et al., 2008) to build sparse partial correlation networks between working memory, oculomotor (RQA), and decision-making (DDM) metrics. Joint networks were estimated via EBICglasso in the bootnet R package (Epskamp et al., 2018) for different participant groups, with variables z-standardized and age regressed out using ordinary least squares regression. Absence of edges indicates associations fully explained by other variables.

Networks were represented as weighted undirected graphs (edge weights = regularized partial correlations). Data were nonparanormal-transformed using huge.npn (huge package) prior to estimation. Communities were detected via Clique Percolation Method with optimal k/I from maximum signed fuzzy modularity. The Minimum Spanning Tree (MST) extracted the core structural skeleton.

Centrality stability was assessed via case-wise bootstrapping Correlation Stability (CS) coefficients (*r* ≥ .70 threshold; higher = more stable). Node strength was calculated as the sum of absolute incoming partial correlations.

Follow-up networks incorporated clinical predictors (age of onset, treatment duration, remission, surgical history), with age regressed out, to examine interactions with cognitive/oculomotor systems.

## Results

### ANCOVA Results for Spatial Span, Drift Diffusion Model, and RQA Parameters

Descriptive statistics for each of the parameters were calculated separately for SSP, DDM, and RQA measures in the two groups shown in *Table 2*.

An analysis of covariance (ANCOVA) was conducted to test for mean differences between patients and control groups. Three-way ANCOVAs with 2 (gender) by 2 (Group) by age design were fitted. The results of the analysis are presented in the combined table (Table 3). Age was included as a covariate.

**Table 2:** Descriptive Statistics for SSP, DDM and RQA Measures in the Two Groups

**Variable**	**Mean (*SD*)**	**Mean (*SD*)**	
		**Patient group**	**Control group**
Span length	6.032(1.55)	5.86 (1.48)	6.22 (1.62)
Attempts	1.03 (.18)	1.03 (.17)	1.033 (.18)
Total errors	14.18 (6.66)	14.88 (6.2)	13.42 (7.1)
Mean time last response	3,938.095 (1,020.89)	4,068.455 (1,174.41)	3,794.7 (805.42)
Drift	.1 (.07)	.08 (.06)	.12 (.07)
Bound	2.91 (.36)	2.96 (.33)	2.86 (.39)
Nondectime	.93 (.10)	.94 (.08)	.92 (.12)
Mean of recurrence rate	.04 (.04)	.03 (.03)	.05 (.04)
Standard deviation of recurrence rate	.02 (.02)	.02 (.02)	.03 (.02)
Mean of laminarity	.87 (.08)	.87 (.06)	.86 (.09)
Standard deviation of laminarity	.06 (.05)	.05 (.03)	.07 (.05)
Mean of entropy of diagonal lines	2.34 (.43)	2.27 (.37)	2.41 (.47)
Standard deviation of entropy of diagonal lines	.36 (.19)	.32 (.16)	.41 (.2)

*Note: Nondectime = non-decision time.*

To account for potential confounding effects of gender and age, an ANCOVA was performed to compare span length between children in the PFT group and the control group.

For Spatial Span (SSP) performance, significant small-to-moderate effects were observed for gender (*p =* .042, η^2^= .034) and age (*p =* .003, η^2^= .071) on span length, suggesting that these demographic factors substantially contribute to individual differences in working memory span. Conversely, neither group membership (*p =* .277, η^2^= .010) nor group × gender interaction (*p =* .692, η^2^= .001) significantly influenced span length. Regarding the number of attempts, a small yet significant group × gender interaction emerged (*p =* .043, η^2^= .033), indicating subtle, sex-dependent variations in how children engaged with the SSP task.

Other SSP measures, including total errors, and reaction times, revealed no significant group or demographic differences, except for mean time to last response, which showed a minor but significant effect of age (*p =* .040, η^2^= .034).

Analysis of parameters obtained from DDM indicated a robust main effect of the group on drift rate (*p =* .001, η^2^= .083), reflecting a moderate effect size; specifically, PFT patients showed significantly slower evidence accumulation compared to controls. Additionally, age significantly contributed to drift rate (*p =* .005, η^2^= .063), further emphasizing developmental impacts on cognitive processing speed. Neither gender (*p =* .163) nor group interactions significantly affected DDM decision boundary or non-decision time.

**Table 3:** ANCOVA Results

		**Group**		**Gender**		**Age**		Group*	**Gender**
		**F**	**η^2^p**	**F**	**η^2^p**	**F**	**η^2^p**	**F**	**η^2^p**
	Span length	1.19	.01	4.24*	.03	9.25**	.07	.16	.00
	Attempts	.05	.00	.02	.00	.30	.00	4.18*	.03
SSP	Total errors	1.81	.02	.01	.00	2.37	.02	.01	.00
	Mean response time last	1.84	.02	.33	.00	4.32*	.03	.07	.00
	Drift	10.89**	.08	1.97	.02	8.09**	.06	.09	.00
DDM	Bound	2.13	.02	1.15	.01	.45	.00	.64	.01
	Nondectime	1.76	.01	1.64	.01	.07	.00	.42	.00
	Mean of recurrence rate	10.03*	.08	4.75*	.04	22.26**	.16	.94	.01
	Standard deviation of recurrence rate	9.03*	.07	2.43	.02	17.35**	.13	.05	.00
	Mean of laminarity Standard	.40	.00	.55	.01	1.55	.01	.18	.00
RQA	deviation of laminarity	4.88*	.04	.76	.01	7.77*	.06	.73	.01
	Mean entropy of of diagonal lines	3.22	.03	.20	.00	2.06	.02	.02	.00
	Standard deviation entropy of of diagonal lines	5.91**	.05	.08	.00	1.13**	.08	.02	.00

Note. Level of significance: * p < .05, ** p < .01

Analysis of parameters obtained from RQA yielded prominent differences in mean recurrence rate (RR mean), with significant effects observed for group (*p =* .002, η^2^= .077), gender (*p =* .031, η^2^= .038), and notably age (*p* < .001, η^2^= .155), underscoring a strong developmental component. Similar moderate effects emerged for RR variability (RR SD), with significant influences of group (*p =* .003, η^2^= .070) and age (*p* < .001, η^2^= .126).

No significant group or demographic effects were observed for mean values of laminarity, and entropy (all p > .20; η^2^ < .02). However, their temporal variability (*SD* values) showed meaningful differences: laminarity *SD* varied significantly by group (*p =* .029, η^2^= .039) and age (*p =* .006, η^2^= .061); entropy *SD* showed significant effects of group (*p =* .017, η^2^= .047) and age (*p =* .002, η^2^= .078); Overall, these findings suggest that temporal variability in gaze behavior is particularly sensitive to both clinical factors and developmental stage, even when mean values remain stable.

### Network Analysis Results

Before constructing the network models, we conducted a series of preliminary correlation analyses to evaluate whether a network approach was warranted and to determine which variables should be included. Specifically, we examined pairwise associations among all cognitive and oculomotor measures using Spearman correlations, computed separately for the control and clinical groups. In this step, we also assessed correlations with age to identify variables substantially shaped by developmental effects. Because age showed widespread associations across domains, we treated it as a confounding factor and removed its influence prior to network estimation. The results are provided in the *Appendix, Tables 1-2*.

We additionally explored associations between the cognitive–oculomotor indicators and clinical variables, including age at disease onset and remission duration, to determine which clinical factors should enter the network model. Only remission duration (rho = -.33, *p* < .01) demonstrated consistent and interpretable associations with core gaze-related metrics (entropy (*SD*)), and was therefore included as the sole clinical variable in the patient network.

Then we constructed separate networks for the control and patient groups, initially including age as a factor due to its significant influence on all measures. Following the guidelines by Epskamp et al. (2018), the Correlation Stability (CS) coefficient indicates how much of the sample can be dropped while retaining a correlation of at least .70 between original and bootstrapped centrality estimates. CS values above .50 are considered good, values above .25 are acceptable, and values below .25 indicate that centrality indices are unreliable. In our analysis, the clinical network reached a CS = .45, falling within the acceptable range and indicating reasonably robust centrality estimates. By contrast, the control network showed CS = .28, just above the minimum acceptable threshold, suggesting that although interpretable, its centrality structure is more sensitive to sampling variation. This pattern indicates greater structural consistency in the clinical group and higher variability in the normative network.

**Figure 1.**
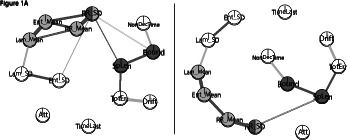
*A.* Network (left ) and corresponding MST (right) for the control group with age effect removed.

The constructed network for control and patient groups without age effects is presented in *Figure 1*.>

The network analysis of the control group reveals a highly structured and modular organization, characterized by two distinct, functionally specialized clusters. The first green cluster included cognitive measures from the Spatial Span (SSP) task - span length (SpLen), along with core Drift Diffusion Model (DDM) parameters such as decision boundary (Bound) and standard deviation of recurrence rate (RR SD) from oculomotor parameters. Notably, some SSP test indicators and the DDM model indicators turned out to be related, but were not included in the cluster. Furthermore, behavioral measures such as the number of attempts and the mean time for last responses in the SSP test were separate and showed no significant connections to other variables. The strong positive connection between Bound (decision threshold) and Span Length (performance) highlights a successful speed-accuracy tradeoff, where a more cautious strategy leads to better outcomes. Basically, the Bound is how our brain shows a plan in thinking, and this is linked to the brain’s stability, measured by recurrence rate (RR SD). Together, they help achieve better high-level thinking, like longer span in memory tasks. Also, the clear link among these three elements in cluster shows an important idea that successful working memory performance is not just a cognitive act, it is a psychophysiological state supported by a specific neural dynamic.

**Figure:**
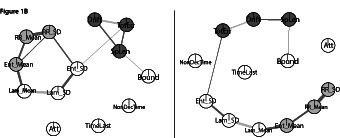
*B.* Network (left ) and corresponding MST (right) for the group of patients with age affect removed.

**Figure:**
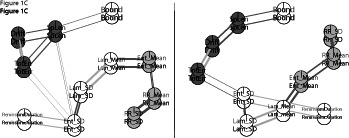
*C.* Network (left ) and corresponding MST (right) for the group of patients with age affect removed with clinical factors.

*Notes. Different colors represent the membership of variables to specific clusters; thicker lines between variables indicate a stronger connection; color of lines indicates direction of correlation between variables — green positive and red negative. Abbreviations: Drift — drift rate; Boundary — decision boundary; Non-DecTime — non-decision time; SpLen— span length of SSP test; Att — number of attempts in SSP test; TotErr — total errors of SSP test; TimeLast — mean time of last response in SSP test; RR Mean — mean of recurrence rate; RR SD — standard deviation of recurrence rate; Lam Mean — mean of laminarity; Lam SD — standard deviation of laminarity; Ent Mean — mean of entropy; Ent SD — standard deviation of entropy; Remission duration — duration of remission*

The second cluster (red) contains eye movement parameters (RQA) such as mean and standard deviation of recurrence rate with mean of entropy (Ent Mean) and mean of laminarity (Lam Mean). This combination points to a unified functional module in the brain’s oculomotor system that governs the stability, complexity, and predictability of visual processing. This cluster shows that this brain system is not fixed. It naturally varies between stability and change. This flexibility is significant for adapting, like smoothly shifting focus from one object to quickly exploring the surroundings. Basically, it represents the main brain activity needed for effective visual gathering of information. By analyzing the network structure, the most influential nodes were identified. Results are provided in the *Appendix*, *Tables 3–5*. The analysis included key metrics such as the mean entropy (.875), the mean recurrence rate (.789), and the standard deviation of the recurrence rate (.782). These measures help evaluate the complexity, stability, and variability of the system, allowing us to determine which nodes play crucial roles within the network. The MST confirms this modular organization segregation of cognitive and eye-movement variables, where the standard deviation of recurrence rate acts as a link between task performance and eye movement. As in the main network, indicators of behavioral measures such as the number of attempts and the mean time for last responses in the SSP test were separate and showed no significant connections to other variables.

We also constructed a network for a group of patients without age effects. Figure 1B shows that the network also has two clusters as in the control group, but it is formed with different parameters.

Analyzing the clinical group, we found a network structure that differs from the control group. The first cluster (green), similar to the control group, included cognitive and DDM parameters, but did not contain indicators from RQA. Besides Span Length, which was also present in the control group, this cluster also included Total Errors and Drift. This suggests that cognitive and oculomotor processes are more disconnected and operate independently. We observed only a weak connection with the standard deviation of the entropy which reflects the system’s dynamic complexity. Similar to the control group, the number of attempts and the mean response time at the end of the SSP test were isolated and showed no significant links to other variables, including non-decision time.

The second cluster (red) included eye movement parameters from RQA, such as the mean and standard deviation of the recurrence rate, along with the mean entropy (Ent Mean). However, it did not include the mean of laminarity (Lam Mean), which was present in the control group. While Lam Mean still relates to other oculomotor measures, it no longer forms part of a united neurodynamic pattern. Instead, it may operate independently, potentially manifesting as maladaptive “stuckness” or, conversely, an inability to stay focused. In this group, the most influential nodes were the standard deviation of the recurrence rate (.918), the mean recurrence rate (.893), and the mean entropy (.875). Notably, the Recurrence Rate *SD* became the most central node in the entire network, signaling a shift toward increased instability in neurodynamic states. This shift implies that the system is dominated by fluctuations and inconsistencies rather than stable average properties. The higher overall strength of variability metrics, such as Recurrence Rate *SD* and mean entropy, suggests that the system is primarily driven by instability, which may also lead to increased rigidity. The MST analysis of the clinical group shows a less resilient, more linear network structure. The weak link between cognitive and oculomotor parameters is confirmed by the standard deviation of entropy, which indicates how well the brain’s control systems manage transitions between cognitive states.

Group comparisons using the Network Comparison Test (NCT) did not yield statistically significant differences in either global strength or overall network structure. Although this result suggests broad similarity between the two networks, it should be interpreted with caution. Prior work indicates that the NCT is sensitive to sample size and may have limited statistical power in cases of small groups or subtle differences, increasing the likelihood of false-negative outcomes (van Borkulo et al., 2023; Borsboom at al., 2021). Given these methodological considerations, the absence of significant findings should not be taken as strong evidence for network equivalence. Instead, the present comparisons are best viewed as exploratory, offering preliminary indications rather than definitive conclusions about potential similarities or differences between groups.

An additional network analysis incorporating the clinical variable of remission duration was performed for the patient group, again controlling for age. Before estimating this model, isolated nodes with no stable connections (*e.g.*, error-related indicators) were removed to ensure a coherent network structure. The resulting clinical–remission network showed moderate stability (CS = .35), which was lower than the stability observed in the age-only clinical model.

The constructed network for a group of patients without age effects with clinical factors is presented in *Figure 1C*.

The resulting network structure was similar to the previous patient-only network, maintaining two clusters. The clinical factor — duration of remission — was negatively associated with standard deviation of entropy. Longer clinical remission was associated with lower variability (more stability) in the entropy over time. Conversely, shorter or less stable remission was linked to less stability in entropy. In this network, the most influential nodes were the standard deviation of the recurrence rate (.973), the mean recurrence rate (.944), and the mean entropy (.919). Similar results were obtained in the clinical group without assessing the inclusion of remission time indicators. Also, like networks in patients without indicators of remission time, the MST emphasized entropy *SD* as a pivotal connector, linking cognitive and oculomotor clusters and mediating relationships between clinical and gaze dynamics during reading tasks. The negative connection between standard deviation of entropy and duration of remission in MST shows that the consistent, time-related stability of neural complexity — more than just its average level — is a key factor for long-term recovery. This suggests that steady neural dynamics are an important protective factor against relapse, making it a valuable focus for monitoring and treatment.

## Discussion

We examined visuospatial working memory (SSP/DDM), reading gaze dynamics (RQA), and cross-domain organization in pediatric PFT survivors vs. typically developing peers. Goals: to compare memory/decision dynamics, characterize recurrence-based gaze, and explore network associations. Patients showed preserved SSP accuracy (span length, errors; driven by age/gender effects), altered DDM dynamics, and rigid oculomotor patterns. Entropy variability — indexing gaze adaptability across segments (Sokolov et al., 2017) — was reduced, decreasing further with remission duration.

SSP robustness suggests compensatory fronto-parietal support post-treatment (Savage et al., 2007). However, accuracy metrics underestimate alterations by ignoring temporal dynamics, missing latent disruptions masked by compensation (Gao et al., 2024). Temporally resolved models are needed to reveal regulatory changes.

In contrast, dynamic markers from DDM revealed clear group distinctions. Patients showed reduced drift rate, indicating less efficient evidence accumulation, while decision boundary and non-decision time remained comparable across groups. The negative association between drift rate and SSP errors demonstrates that dynamic parameters capture meaningful individual differences even in the absence of accuracy-based group differences. This dissociation between preserved performance and altered temporal dynamics underscores the selective vulnerability of cerebellarsupported timing mechanisms: cerebellar disruption may affect how cognitive operations unfold over time without necessarily degrading overt task outcomes.

RQA revealed clear developmental effects and group differences in the temporal organization of gaze during reading. Recurrence rate, laminarity, and entropy were strongly interrelated across participants, reflecting the structured and temporally stable nature of gaze control. Age was a major predictor of recurrence-related metrics, consistent with the maturation of oculomotor stability throughout childhood and adolescence.

Importantly, group differences emerged not in mean-level values but in variability-based measures. Patients showed reduced variability in laminarity and entropy along with higher recurrence rate and recurrence variability. This pattern reflects more constrained, stereotyped, and less adaptable gaze dynamics. The divergence between mean-level measures (which remained similar) and variability (which strongly differentiated the groups) aligns with theoretical accounts emphasizing that neurodynamic flexibility, not average behavior, carries clinically relevant information after neurological insult (Marusak et al., 2018). Variability captures the system’s capacity to adjust to moment-to-moment demands; reduced variability points to a shift toward rigidity and diminished adaptive modulation. This divergence reinforces the notion that variability-based indices often reveal dysfunction more clearly than mean-level metrics, because they capture the system’s adaptive capacity rather than its average state.

In the context of cerebellar dysfunction, reduced variability and increased recurrence likely represent attempts to stabilize sensory input by narrowing the range of possible oculomotor states. Such compensation may support adequate performance during reading but at the cost of reduced adaptability when processing demands fluctuate. This mechanistic interpretation complements the preserved SSP accuracy despite altered drift -rate dynamics: patients may sustain behavior through stereotyped visuomotor strategies that reduce cognitive load.

Network analysis provided an integrated view of how cognitive and oculomotor processes relate within each group. In typically developing children, SSP and DDM variables formed a cohesive cognitive cluster, reflecting complementary facets of the same underlying working-memory operation. Oculomotor variables formed coherent sub-networks capturing temporal stability (laminarity) and dynamical complexity (entropy, recurrence rate). Centrality estimates identified laminarity and span length as influential nodes, consistent with evidence linking structured gaze behavior to efficient information processing (Ferguson et al., 2021; Rayner et al., 2006).

In patients, networks showed reduced integration: SSP/DDM remained connected (despite altered dynamics), but had weaker, diffuse oculomotor links. Laminarity decoupled from its cluster, entropy had sparse connections, and oculomotor coherence decreased—paralleling group-level variability reductions and cross-domain decoupling. Entropy variability was highly central, bridging cognitive, oculomotor, and clinical nodes.

Decision boundary became isolated, indicating disrupted deliberation-working memory coordination (consistent with preserved accuracy but altered dynamics). Despite separate tasks, joint organization indexes shared mechanisms such as attentional control and cerebellar temporal integration.

Patient networks showed higher stability (CS coefficient), reflecting rigid architecture with less individual variability and narrower configurations (vs. adaptive control networks).

Remission duration predicted lower entropy variability, indicating consolidation of rigid gaze strategies (not flexibility recovery). Entropy variability marks cerebellocortical reorganization (Gao et al., 2024; Kasatkin et al., 2022; Rimrodt et al., 2009); early interventions are needed to restore adaptability.

Together, the findings indicate that pediatric cerebellar injury alters the adaptability and coordination of distributed cognitive–motor systems, rather than producing categorical behavioral deficits. The cerebellum plays a central role in temporal integration across frontal and parietal networks (Strick et al., 2009) involved in attention, working memory, and executive control (Clark et al., 2021; Iosif et al., 2023). Reduced drift rate, decreased variability in gaze metrics, and weaker cognitive–oculomotor coupling are consistent with disruptions to timing-based modulation that normally enables fluid coordination between cognitive processes and gaze control (Kim et al., 2021).

Importantly, these alterations likely reflect broader cerebello–cortical disruption rather than focal cerebellar damage alone (Schmahmann et al., 2007). Posterior fossa tumors and their treatments can affect cerebellar outflow pathways, fronto-parietal control networks, and long-range timing circuits, producing downstream consequences for attentional allocation, working-memory regulation, and oculomotor adaptability (Guell et al., 2018; Stoodley & Schmahmann, 2010). Reduced variability and increased network rigidity may thus reflect compensatory strategies that stabilize performance by narrowing the range of possible cognitive and gaze-control states. Classical accuracy metrics, which index end-point performance rather than temporal dynamics, may fail to detect such adaptations, highlighting the value of dynamic and integrative approaches such as DDM, RQA, and exploratory network methods.

Developmentally, reduced oculomotor flexibility and fragmented cross-domain organization may reflect deviations in the calibration of cognitive–motor systems rather than isolated impairments. The cerebellum contributes to the maturation of distributed neural networks, and early injury may alter the developmental trajectories through which cognitive and oculomotor functions become coordinated. This perspective emphasizes the need to conceptualize pediatric cerebellar injury as a systemic reorganization of cognitive architecture, with potential long-term implications for adaptability and learning (Dalboni da Rocha et al., 2024).

Longitudinal and interventional research will be valuable for determining whether gaze-control flexibility and cross-domain network organization can be enhanced through targeted training, such as visual–motor coordination tasks or dynamic attentional interventions. It will also be important to assess whether entropy variability or network connectivity can serve as prognostic or treatment-responsive indicators. Combining behavioral, neural, and computational tools may help clarify mechanisms underlying reduced dynamical complexity and support the development of individualized rehabilitation strategies.

## Conclusion

Pediatric PFT survivors showed altered cognitive-oculomotor organization vs. controls, despite preserved working memory accuracy. Integrating DDM, RQA, and networks revealed disrupted evidence accumulation, reduced gaze flexibility, and fragmented topology: controls had coherent clusters with entropy as the key hub; patients showed weaker cognitive-oculomotor links and central entropy variability indexing neurodynamic integrity.

Taken together, these findings illustrate the value of moving beyond isolated behavioral outcomes toward temporally resolved and network-oriented approaches capable of detecting subtle disruptions in cognitive organization that may not be apparent in standard performance measures. The prominence of entropy variability as a cross-domain connector suggests promising avenues for future assessment and monitoring efforts, particularly in identifying children who may benefit from interventions aimed at enhancing cognitive flexibility and oculomotor adaptability.

## Limitations

This study has several limitations that warrant consideration. First, the network-based findings should be interpreted as exploratory. Network estimation and comparison procedures are sensitive to modeling choices—including variable selection, regularization, and bootstrapping assumptions—and the resulting structures require replication to establish their robustness. Complementary analytic approaches (*e.g.*, Bayesian networks or dynamical-systems modeling) and independent samples will be important for confirming the stability of these patterns.

Second, the cross-sectional design limits our ability to determine causal relationships or characterize developmental trajectories following cerebellar injury. Longitudinal approaches will be essential for understanding how cognitive–oculomotor coupling evolves over time and how these changes relate to factors such as treatment history and remission duration.

Third, although the clinical group reflects the heterogeneity typical of pediatric posterior fossa tumor survivors—including differences in tumor type, treatment modalities, and disease progression—this diversity may introduce confounding variance. Such heterogeneity increases ecological validity, but may limit the specificity with which particular mechanisms or pathways can be linked to the observed network alterations.

Fourth, although we employed detailed behavioral and computational measures, the absence of neuroimaging data limits the interpretation of the neural substrates underlying the observed cognitive–oculomotor reconfiguration. Structural or functional imaging could help clarify whether altered interactions reflect disruptions within cerebellar–cortical pathways or more widespread changes in distributed networks.

A further limitation stems from the lack of eye-tracking data during the working memory task. While reading-based eye-movement measures provide rich dynamical information, the inability to monitor gaze behavior during the SSP task constrains our understanding of how oculomotor patterns contribute to, or compensate for, working-memory demands. Integrating eye-tracking into memory tasks in future work would help disentangle sources of behavioral variability—such as fluctuations in attention, encoding efficiency, or strategy use—and determine whether similar organizational patterns emerge outside the reading context.

Finally, we did not collect information on participants’ engagement in cognitive rehabilitation, educational supports, medication use, or socioeconomic factors. These contextual variables may shape both developmental outcomes and the compensatory strategies children employ to manage cognitive demands. Their inclusion in future research would provide a more comprehensive understanding of individual differences in recovery and adaptation.

## Figures and Tables

**Table 1:** Correlations for Control Group (Age Variable Removed)

** **	**Age**	**SpLen**	**Att**	**TotErr**	**Time-Last**	**drift**	**boundary**	**NonDec Time**	**RR Mean**	**RR Mean**	**Lam Mean**	**Lam SD**	**Ent Mean**
Age	1.00	–.02	.61***	.04	.07	.49***	.02	.36**	.43***	.18	.16	.32*	.40**
SpLen	–.02	1.00	–.05	–.02	–.15	–.15	–.07	–.11	–.02	.13	–.18	.05	–.04
Att	.61***	–.05	1.00	.16	–.55***	.34**	.07	.31*	.33**	.18	.02	.28*	.18
TotErr	.04	–.02	.16	1.00	–.21	.30*	.22	–.13	–.08	.04	–.02	.04	–.03
TimeLast	.07	–.15	–.55***	–.21	1.00	–.10	–.04	–.09	–.11	–.11	.13	–.06	.05
Drift	.49***	–.15	.34**	.30*	–.10	1.00	–.09	.30*	.32*	.16	–.04	.23	.23
boundary	.02	–.07	.07	.22	–.04	–.09	1.00	–.14	–.05	–.23	.32*	–.20	–.02
NonDecTime	.36**	–.11	.31*	–.13	–.09	.30*	–.14	1.00	.88***	.73***	–.31*	.81***	.20
RR Mean	.43***	–.02	.33**	–.08	–.11	.32*	–.05	.88***	1.00	.58***	–.09	.68***	.42***
RR SD	.18	.13	.18	.04	–.11	.16	–.23	.73***	.58***	1.00	–.68***	.88***	–.12
Lam Mean	.16	–.18	.02	–.02	.13	–.04	.32*	–.31*	–.09	–.68***	1.00	–.48***	.47***
Lam SD	.32*	.05	.28*	.04	–.06	.23	–.20	.81***	.68***	.88***	–.48***	1.00	.13
Ent Mean	.40**	–.04	.18	–.03	.05	.23	–.02	.20	.42***	–.12	.47***	.13	1.00

Notes. Level of significance: * p < .05, ** p < .01. Abbreviations: Drift - drift rate; boundary - decision boundary; NonDecTime - non-decision time; SpLen - span length of SSP test; Att - number of attempts in SSP test; TotErr - total errors of SSP test; TimeLast - mean time of last response in SSP test; RR Mean- mean of recurrence rate; RR SD - standard deviation of recurrence rate; Lam Mean - mean of laminarity; Lam SD - standard deviation of laminarity; Ent Mean - mean of entropy ; Ent SD - standard deviation of entropy.

**Table 2:** Correlations for Patients’ Group with Clinical Factors (Age Variable Removed)

** **	**Age**	**SpLen**	**Att**	**TotErr**	**Time-Last**	**drift**	**boun-dary**	**NonDec Time**	**RR Mean**	**RR Mean**	**Lam Mean**	**Lam SD**	**Ent Mean**	**Age onset**	**Treat-ment months**	**Remis-sion months**
Age	1.00	–.12	.42***	–.10	.28*	.23	–.04	.24	.19	.00	.13	.11	.24*	.10	.03	–.17
SpLen	–.12	1.00	–.02	–.14	–.02	.03	.44***	.17	.08	.03	–.03	.06	–.10	–.10	.07	.11
Att	.42***	–.02	1.00	–.06	–.47***	.15	.01	.17	.21	.11	.18	.20	.26*	.10	.17	–.10
TotErr	–.10	–.14	–.06	1.00	.00	.11	.02	.09	.14	.04	–.03	.10	–.06	.11	–.10	–.06
TimeLast	.28*	–.02	–.47***	.00	1.00	–.16	–.15	.03	–.02	–.09	–.02	–.10	.06	–.11	–.13	.07
Drift	.23	.03	.15	.11	–.16	1.00	.03	–.03	.01	.07	.01	.02	.01	.08	.18	–.15
boundary	–.04	.44***	.01	.02	–.15	.03	1.00	.13	.06	–.04	.00	–.01	–.09	–.04	–.07	.07
NonDecTime	.24	.17	.17	.09	.03	–.03	.13	1.00	.92***	.41***	.14	.72***	.51***	.12	–.17	–.08
RR Mean	.19	.08	.21	.14	–.02	.01	.06	.92***	1.00	.34**	.25*	.65***	.58***	.22	–.13	–.16
RR SD	.00	.03	.11	.04	–.09	.07	–.04	.41***	.34**	1.00	–.68***	.77***	–.05	–.17	.12	.15
Lam Mean	.13	–.03	.18	–.03	–.02	.01	.00	.14	.25*	–.68***	1.00	–.30*	.55***	.24	–.08	–.22
Lam SD	.11	.06	.20	.10	–.10	.02	–.01	.72***	.65***	.77***	–.30*	1.00	.32**	–.05	–.01	.05
Ent Mean	.24*	–.10	.26*	–.06	.06	.01	–.09	.51***	.58***	–.05	.55***	.32**	1.00	.27*	.05	–.33**
Age onset	.10	–.10	.10	.11	–.11	.08	–.04	.12	.22	–.17	.24	–.05	.27*	1.00	–.01	–.92***
Treatment months	.03	.07	.17	–.10	–.13	.18	–.07	–.17	–.13	.12	–.08	–.01	.05	–.01	1.00	–.25*
Remission months	–.17	.11	–.10	–.06	.07	–.15	.07	–.08	–.16	.15	–.22	.05	–.33**	–.92***	–.25*	1.00

Notes. Level of signi! cance: * p < .05, ** p < .01. Abbreviations: Drift - drift rate; boundary - decision boundary; NonDecTime - non-decision time; SpLen - span length of SSP test; Att - number of attempts in SSP test; TotErr - total errors of SSP test; TimeLast - mean time of last response in SSP test; RR Mean - mean of recurrence rate; RR SD - standard deviation of recurrence rate; Lam Mean - mean of laminarity; Lam SD - standard deviation of laminarity; Ent Mean - mean of entropy; Ent SD - standard deviation of entropy ; AgeOnset - age at disease onset; Treatment months - duration of treatment; remission months – duration of remission.

**Table 3:** Strength of Nodes for Control Group

**Node**	**Strength**
Bound	.46
Laminarity *SD*	.39
Span length	.58
Entropy *SD*	.32
Nondectime	.20
Laminarity mean	.77
Attempts	.00
Mean time last response	.00
Entropy mean	.87
Recurrence rate mean	.79
Recurrence rate *SD*	.78
Drift	.27
Total errors	.47

**Table 4:** Strength of Nodes for Patient Group without Clinical Factors

**Node**	**Strength**
Span length	.48
Recurrence rate mean	.89
Entropy *SD*	.57
Attempts	.00
Mean time last response	.00
Nondectime	.00
Bound	.05
Recurrence rate *SD*	.92
Entropy mean	.88
Laminarity *SD*	.67
Drift	.48
Total errors	.50
Laminarity mean	.76

**Table 5:** Strength of Nodes for Patient Group with Clinical Factors

**Node**	**Strength**
Span length	.73
Recurrence rate mean	.94
Laminarity *SD*	.75
Entropy *SD*	.90
Remission months	.20
Recurrence rate *SD*	.97
Bound	.10
Entropy mean	.92
Laminarity mean	.86
Drift	.64
Total errors	.72
